# Purification and Partial Characterization of Bacteriocin Lac-B23, a Novel Bacteriocin Production by *Lactobacillus plantarum* J23, Isolated From Chinese Traditional Fermented Milk

**DOI:** 10.3389/fmicb.2018.02165

**Published:** 2018-10-01

**Authors:** Jianming Zhang, Yanyan Yang, Hui Yang, Yushan Bu, Huaxi Yi, Lanwei Zhang, Xue Han, Lianzhong Ai

**Affiliations:** ^1^Department of Food Science and Engineering, Harbin Institute of Technology, Harbin, China; ^2^College of Food Science and Engineering, Ocean University of China, Qingdao, China; ^3^Shanghai Engineering Research Center of Food Microbiology, University of Shanghai for Science and Technology, Shanghai, China

**Keywords:** bacteriocin Lac-B23, food industry, *Lactobacillus plantarum*, antimicrobial activity, characterization, purification

## Abstract

The exploration and evaluation of bacteriocin-producing lactic acid bacteria (LAB) have been one of the powerful means to food preservation. A total of 300 strains were isolated from Chinese traditional fermented milk products. A bacteriocin-producing LAB, named *Lactobacillus plantarum* J23, was screened and identified. Bacteriocin Lac-B23 from *L. plantarum* J23 was purified by 80% ammonium sulfate precipitation, cation-exchange chromatography, and reverse-phase high-performance liquid chromatography. Molecular weight of bacteriocin Lac-B23 was determined to be approximately 6.73 kDa by tricine sodium dodecyl sulfate-polyacrylamide gel electrophoresis analysis, and it was confirmed as a novel bacteriocin by liquid chromatography-mass spectrometry. Moreover, bacteriocin Lac-B23 showed thermal stability when heated at below 100°C for 30 min, pH stability between pH 2.0 and 12.0, and sensitivity to trypsin, proteinase K, and proteinase E. The antimicrobial activity of bacteriocin could be enhanced by addition of Fe^2+^, Mn^2+^, and ethyl alcohol, and inhibited by Cu^2+^, K^+^, Ca^2+^, Zn^2+^, Mg^2+^, and sodium chloride. The results suggested bacteriocin Lac-B23 to have potential application prospects in the food industry.

## Introduction

Bacteriocins, defined as biologically active proteins or peptides, are found in almost every bacterial species. Bacteriocins from lactic acid bacteria (LAB), especially, have drawn special attention due to their potential application as natural food preservatives in the food industry (Udhayashree et al., 2012; Gálvez et al., 2014; Ramu et al., 2015). At present, according to the classification scheme of Klaenhammer (Klaenhammer, 1993; Rea et al., 2011). LAB bacteriocins can be divided into four classes: class I (lantibiotics), containing the unusual amino acid lanthionine, dehydrated residues, and β-methyllanthionine; class II, containing small heat-stable non-lanthionine peptides (<10 kDa) and class III, containing large heat-labile protein (>30 kDa); and class IV, containing complex proteins with lipid or carbohydrate moieties. Out of these, only nisin (Class I) has been produced industrially. However, since nisin could only inhibit gram-positive bacteria (Deegan et al., 2006), mining for novel bacteriocins with a broad antibacterial spectrum has been constantly highlighted. A lot of traditional fermented food is used in China, including fermented vegetables, fermented meat, and fermented milk. These traditional fermented foods contain a variety of the microbial community (such as lactobacilli, lactococci, and yeast) depending on the specific environment (Li et al., 2012; Chen et al., 2014; Lü et al., 2014a). In addition, some bacteria in these fermented foods can produce antibiotic substances, like bacteriocins, which can inhibit the growth of food-borne spoilage and pathogenic bacteria; hence, they can contribute to the long shelf-life of fermented food (Balciunas et al., 2013). For example, Luo et al. (2011) screened the bacteriocin-producing LAB from kurut, which could inhibit the growth of *Staphylococcus aureus* and *Escherichia aerogenes*. Bacteriocin F1, produced by *Lactobacillus paracasei* subsp. tolerans FX-6, was purified from Tibetan kefir, and exhibited antifungal activity (Miao et al., 2014).

Three hundred bacterial strains had been isolated previously from Chinese traditional fermented milk (Yi et al., 2011). The study aimed to screen the bacteriocin-producing strain and purify and characterize the bacteriocin, which would provide fundamental information about its potential and facilitate further development.

## Materials and Methods

### Bacteriocin Activity Assay

The activated strains were inoculated in fresh De Man, Rogosa, and Sharpe agar (MRS), containing the following constituents per liter: 20 g glucose, 5 g yeast extract, 10 g peptone, 7.5 g beef powder, 2 g dipotassium phosphate, 2 g ammonium citrate dibasic, 0.25 g manganese sulfate, 0.58 g magnesium sulfate, 1 mL tween-80, and 5 g sodium acetate at 37°C for 16 h, centrifuged at 8000 rpm for 3 min. The supernatants were adjusted to pH 6.0–6.5 with 1 M NaOH, and filtered through a 0.22 μm filter (Millipore, United States). These supernatants were called “cell-free supernatants containing bacteriocin” (CFSCB). Bacteriocin activity tests were performed by agar well diffusion assay with some modifications (Parente et al., 1995). Fifteen milliliters of 1.5% (w/v) agar were poured into a sterile plate as base medium, and 7.8 mm Oxford cups were put ontop. Molten-soft MRS broth agar (0.6%, w/v) was inoculated with 10 μL indicator strain (10^8^CFU/mL). About 15 mL of the inoculated soft agar were poured onto the surface of bottom medium. After solidification, the Oxford cups were pulled out and 100 μL of CFSCB was placed into the 7.8-mm well. The plates were incubated at 37°C, and growth-inhibition zones were measured after 12 h. One bacteriocin unit was arbitrarily defined as the reciprocal of the highest dilution that showed a clear inhibition zone and was expressed as activity units (AU) per mL. Protein concentration was estimated using Pierce BCA Protein Assay Kit (Thermo Fisher Scientific).

### Screening and Identification of Bacteriocin-Producing LAB

Three hundred strains were isolated previously from Chinese traditional fermented milk in the Xinjiang, Gansu, and Qinghai provinces. All isolates were grown in MRS at 37°C for 24 h. Antimicrobial activity was determined by the agar well diffusion assay as described earlier. *Listeria monocytogenes*, as indicator bacteria, were grown in brain heart infusion (BHI) broth (Oxoid, United Kingdom). In order to eliminate the effect of hydrogen peroxide, antimicrobial activity was tested after treatment of supernatants with catalase using agar well diffusion assay.

Identification of strain was assayed by 16S rDNA test and using API50 CHL V5.1. Genomic DNA was extracted using the bacterial DNA isolation kit (Omega, BioTek), and 16S rDNA gene was amplified using universal prokaryotic primers (forward primer, 5′-AGAGTTTGATCCTGGCTCAG-3′ and reverse primer, 5′-CTACGGCTACCTTGTTACGA-3′) (Amarila et al., 2009). A 25 μL of polymerase chain reaction (PCR) contained Taq polymerase 0.25 μL, 10 × Taq buffer 2.5 μL, dNTP 2.0 μL, each primer 2.5 μL, MgCl_2_ 2.5 μL, DNA template 2.5 μL, and dd-H_2_O 10.25 μL. The PCR amplification program was performed in a PCR thermocycler (Applied Biosystems) as follows: predenaturation at 94°C for 5 min; 30 cycles of denaturation at 94°C for 1 min, annealing at 55°C for 1 min, extension at 72°C for 2 min; and final extension at 72°C for 2 min. Amplified 16S rDNA gene fragment was assayed using 1% (w/v) agarose gel electrophoresis and was sequenced by Sangon Biotech, China. Finally, the 16S rDNA gene sequence was determined from GenBank database, and phylogenetic analysis was performed by MEGA 5.0.

### Purification of Bacteriocin

Bacteriocin-producing LAB was inoculated in 1 L MRS and incubated at 37°C for 16 h. Cells were discarded by centrifugation at 8,000 rpm for 3 min. Bacteriocin present in the cell-free supernatant was subsequently precipitated by ammonium sulfate, after which the precipitates were collected by centrifugation at 10000 rpm for 20 min and dissolved in double distilled water. The dissolved bacteriocin was filtered through a 0.22-μm filter and dialyzed using a 1000 Da cut-off membrane (Solarbio, China). The dialyzed fraction, as fraction I, was tested for antimicrobial activity by agar well diffusion assay. The AKTA purifier (GE Healthcare, Uppsala, Sweden), equipped with SP Sepharose Fast Flow column, was used for further purification of fraction I. The system was equilibrated with 50 mmol/L sodium phosphate buffer (pH 6.0), and the column was eluted with 50 mmol/L sodium phosphate buffer, containing 1 mol/L NaCl, at a flow rate of 2 mL/min. The elution procedure was monitored by a UV detector at 220 nm. Fractions with antimicrobial activity were pooled and concentrated by a lyophilizer. The active fraction II was finally purified by reverse-phase high-performance liquid chromatography (RP-HPLC) (Agilent) using C-18 reverse-phase column. Elution program was as follows: an initial gradient from 0 to 60% of solvent B (0.1% acetic acid in acetonitrile) from 0 to 20 min, followed by a gradient from 60 to 100% of solvent B from 20 to 25 min, and finally using 100% solvent B for 5 min. The flow rate was 1 mL/min and elution was monitored by a UV detector at 220 nm. The fractions were collected and analyzed for antimicrobial activity.

### Tricine SDS–PAGE Analysis

Tricine SDS–PAGE analysis of purified bacteriocin was performed with 16% resolving gel and 4% stacking gel. The gel was cut into two parts: (i) gel was stained with Coomassie brilliant blue R-250 in order to determine the molecular size of bacteriocin and (ii) another gel was fixed in 20% (v/v) isopropanol and 10% (v/v) acetic acid for 2 h, and rinsed in sodium phosphate buffer (pH 6.0) for 1 h. After that, the gel was overlaid with soft agar medium inoculated with the indicator strain for 48 h.

### Liquid Chromatography Mass Spectrometry Analysis

Bacteriocin was identified by liquid chromatography–mass spectrometry (LC-MS), using active band of tricine SDS–PAGE gel at Shanghai Applied Protein Technology Co., Ltd., in China.

### Effect of Heat, pH, Enzymes, Metal Ion, Ethyl Alcohol, and Sodium Chloride on Bacteriocin Activity

Heat treatment of bacteriocin was conducted at 25, 30, 37, 50, 60, 70, 80, 90, 100, and 121°C for 30 min. The pH sensitivity of bacteriocin was performed at pH of 2.0, 4.0, 5.0, 6.0, 7.0, 8.0, 9.0, 10.0, and 12.0 for 2 h at 37°C. After the pH treatment, pH was adjusted to 6.0–6.5. Sensitivity to enzymatic proteolysis was tested by treatment of bacteriocin with trypsin, proteinase K, proteinase E, papain, and pepsin (1.0 mg/mL) at 37°C for 2 h. To test the influence of metal ion, bacteriocin was treated with KCl, CaCl_2_, FeSO_4_, ZnSO_4_, CuCl_2_, MgSO_4_, and MnSO_4_ (2.0 mol/L) for 24 h at 37°C. To study the effect of ethyl alcohol, different concentrations of ethyl alcohol (2, 4, 6, 8, 10, and 12%) were added to bacteriocin aliquots at 37°C. To investigate the effect of sodium chloride, different concentrations of sodium chloride (2, 4, 5, 6, 7, 8, 10 and 12%) were added to bacteriocin aliquots at 37°C. Antimicrobial activity of all the treated bacteriocins was determined by agar well diffusion assay as described earlier.

### Statistical Analysis

All data are expressed as the mean of three independent experiments. All statistical analyses were performed with Origin 8.6 and SPSS 19.0.

## Results and Discussion

### Isolation and Identification of Bacteriocin-Producing Strain

A total of 300 strains were isolated and screened for bacteriocin-producing strains. After eliminating effects of hydrogen peroxide and organic acid, only the supernatants from six strains (G8L, G16C, G10L, Q12L, YmDZA1-1, and J23) retained antimicrobial activity against *Listeria monocytogenes* (**Table 1**). However, J23 had stronger anti-*L. monocytogenes* activity than the rest five strains. Therefore, strain J23 was used as bacteriocin-producing strain for further research.

**Table 1:** Antimicrobial activity of the primarily selected strains against *Listeria monocytogenes*.

Strains	Anti-*Listeria monocytogenes* activity
G8L	+
G16C	++
G10L	+
Q12L	++
YmDZA1-1	+
J23	+++


*“+++” was inhibition zone >11.00 mm; “++” was inhibition zone from 9.00 to 11.00 mm; “+” was inhibition zone from 7.00 to 9.00 mm.*

**FIGURE 1:**
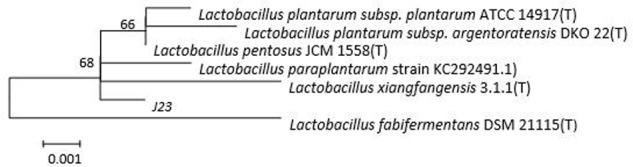
Phylogenetic tree based on the 16S rDNA sequences of strain J23.

The 16s rDNA sequence analysis of strain J23 showed 99% sequence similarity with that of *Lactobacillus plantarum* strain 3m-1 (GenBank accession number KC292491.1) using the BLAST algorithm. A phylogenetic tree was established by sequence alignment (**Figure 1**) and comparison with other LAB. Results were further confirmed by API identification. The strain J23 was named *L. plantarum* J23. According to our knowledge, bacteriocin-producing *L. plantarum* had earlier been isolated from various foods, including wine products (Van Reenen et al., 1998), fermented meat products (Todorov et al., 2010), dairy products (Powell et al., 2007; Xie et al., 2011), and molasse products (Todorov and Dicks, 2005). The *L. plantarum* as a “Generally Recognized As Safe” (GRAS) strain could be applied to many foods, such as fermented berries (Palomino et al., 2015) and refrigerated food (Crowley et al., 2012). Therefore, *L. plantarum* J23 has appreciable potential as bacteriocin-producing strain in the food industry.

### Purification of Bacteriocin

**FIGURE 2:**
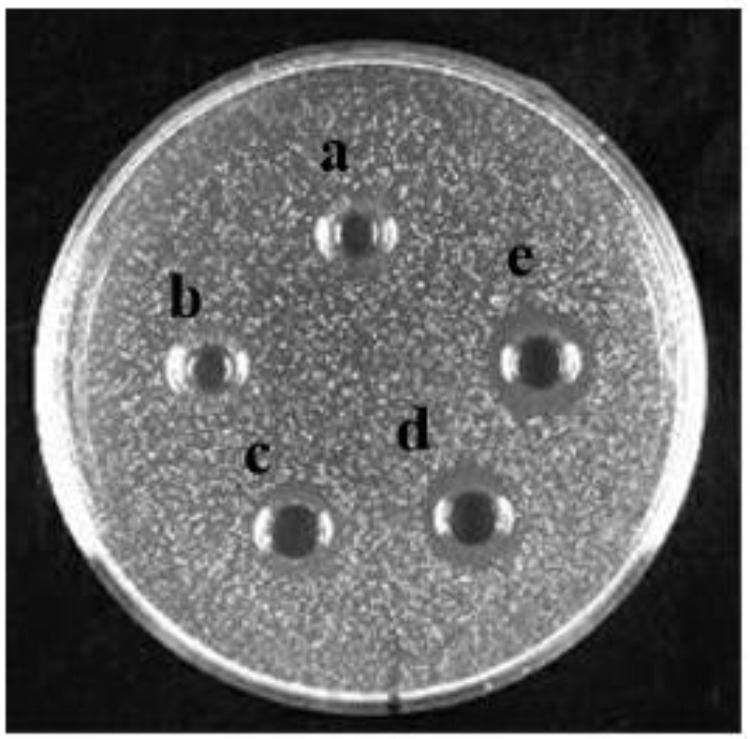
The anti-*Listeria monocytogenes* activity of bacteriocin produced with different ammonium sulfate precipitations: treated with **(a)** 40% ammonium sulfate, **(b)** 50% ammonium sulfate, **(c)** 60% ammonium sulfate, **(d)** 70% ammonium sulfate, and **(e)** 80% ammonium sulfate.

**FIGURE 3:**
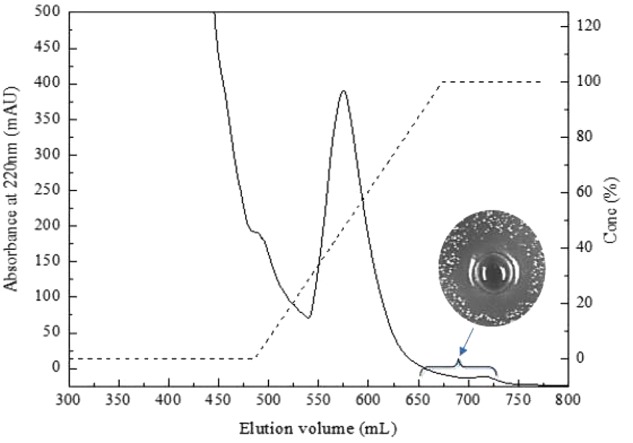
Purification of bacteriocin Lac-B23 using SP Sepharose Fast Flow column.

**FIGURE 4:**
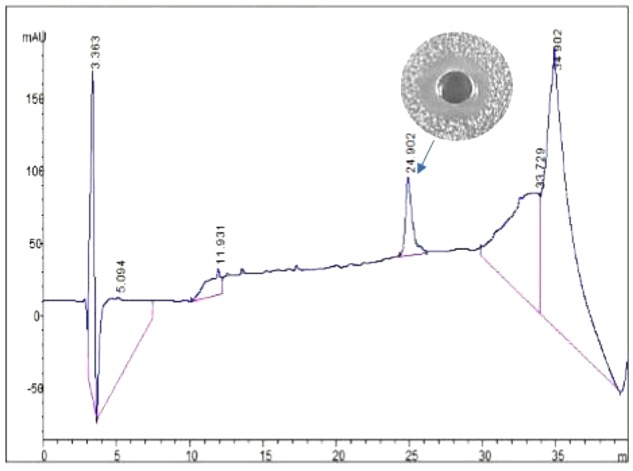
Analytical HPLC chromatography of bacteriocin Lac-B23.

Bacteriocin produced by *L. plantarum* J23 was purified via a three-step procedure, including ammonium sulfate precipitation, cation-exchange chromatography, and RP-HPLC. Initially, the cell-free supernatant containing bacteriocin was precipitated by 40, 50, 60, 70, and 80% ammonium sulfate. The results showed that 80% ammonium sulfate enhanced the antimicrobial activity by 10.31-fold with a 48% yield (**Figure 2** and **Table 2**). This illustrated that 80% ammonium sulfate could be used as a crude method of bacteriocin purification, consistent with results from other research groups (Messaoudi et al., 2012; Lü et al., 2014b). The precipitates (by 80% ammonium sulfate) were further purified by cation-exchange chromatography, which revealed that the fractions with antimicrobial activity were collected between 650 and 740 mL (**Figure 3**), although it was at the tail of peak. This might be due to very low concentrations of the protein. However, the antimicrobial activity in these fractions was 16374.78 AU/mg of protein, which was an 827.01-fold increase compared with that in the cell-free supernatant (**Table 2**). In the final step, the active fractions II were applied to an RP-HPLC column. A single absorption peak at 220 nm was observed at the retention time 24.90 min (**Figure 4**), and this fraction had an anti-*L. monocytogenes* activity. In conclusion, a pure bacteriocin could be obtained by three-step purification methods (80% ammonium sulfate precipitation, cation-exchange chromatography, and RP-HPLC), and could provide samples for the characterization of bacteriocin.

**Table 2:** Purification of bacteriocin produced by *L. plantarum* B23.

Sample	Volume (mL)	Total protein (mg)	Total bacteriocin activity (AU)	Specific activity (AU/mg)	Purification (fold)	Yield (%)
Cell-free supernatant	1000	8082.21	160000	19.80	1	100
80% ammonium sulfate precipitation	120	376.27	76800	204.11	10.31	48
Cation-exchange chromatography	7.1	1.11	18176	16374.78	827.01	11.36


### Molecular Mass and Identification of Bacteriocin

**FIGURE 5:**
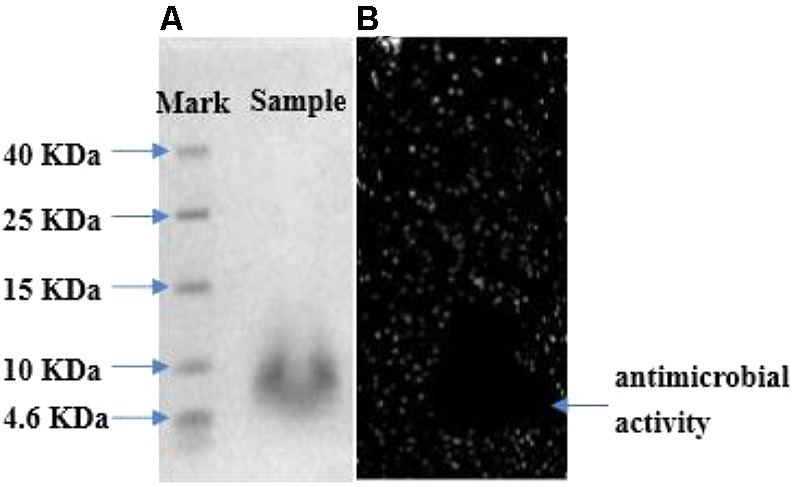
**(A)** Tricine SDS–PAGE analysis of purified bacteriocin Lac-B23; **(B)** gel overlaid.

Tricine SDS–PAGE analysis of fraction II gave a single band between 4.6 and 10 kDa in the stained gel (**Figure 5A**). The molecular mass of the peptide band was found to be approximately 6.73 kDa by Quantity One analysis, which conformed to molecular mass characteristics of class II bacteriocin. In addition, another overlay gel showed a clear antibacterial zone in the same location (**Figure 5B**). Therefore, the single peptide band in the stained gel could be deduced as an antimicrobial peptide. The active band from tricine SDS–PAGE gel was analyzed with LC-MS to identify bacteriocin. The result suggested it to be a novel bacteriocin, named bacteriocin Lac-B23. It could lay the foundation for structure–activity relationship study of bacteriocin Lac-B23 in the future.

### Effect of Heat Treatment, pH, and Enzymes on Bacteriocin Activity

**FIGURE 6:**
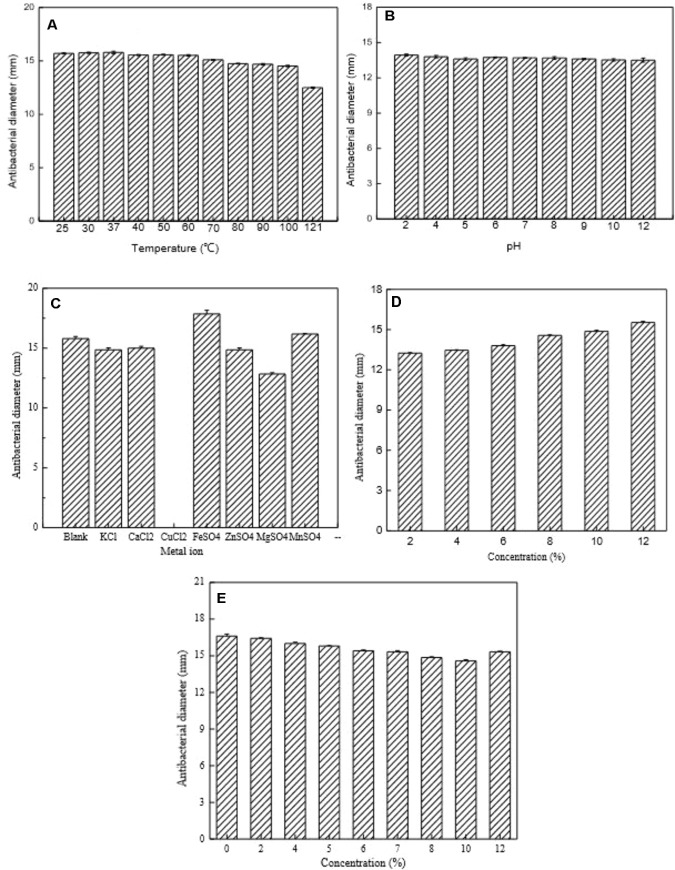
Partial characterization of bacteriocin Lac-B23. **(A)** Effect of heat treatment; **(B)** Effect of pH; **(C)** Effect of metal ion; **(D)** Effect of ethyl alcohol; and **(E)** Effect of sodium chloride, on the antimicrobial activity.

**FIGURE 7:**
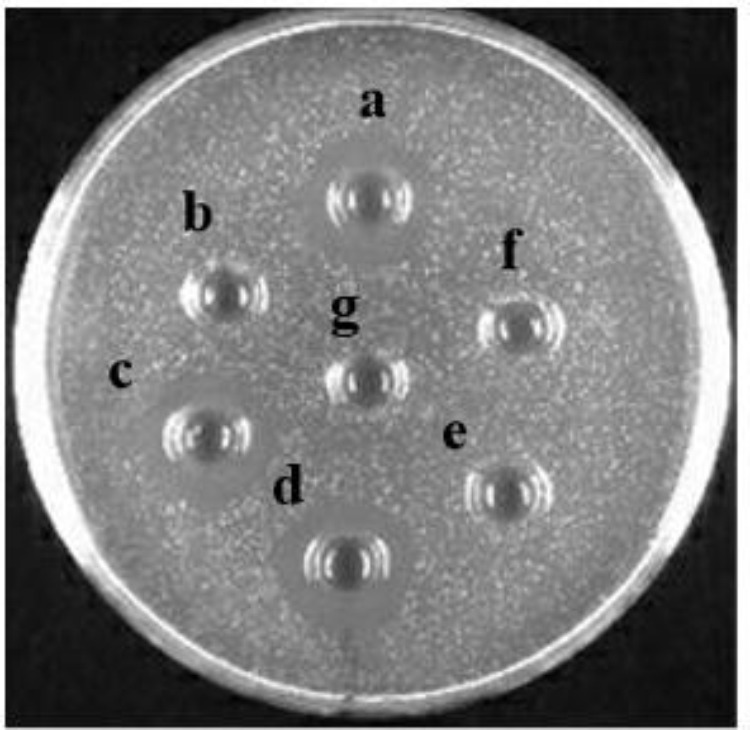
Effect of different proteases on Bacteriocin Lac-B23: **(a)** cell-free supernatants with no treatment; treated with **(b)** proteinase E; **(c)** papain; **(d)** pepsin; **(e)** trypsin; **(f)** proteinase K; and **(g)** blank control.

The influence of heat treatment, pH, and enzymes on bacteriocin activity is shown in **Figures 6A,B, 7**. The antimicrobial activity of bacteriocin Lac-B23 was retained after heat treatment from 25 to 100°C for 30 min; however, the antimicrobial activity was significantly reduced when incubated at 121°C for 30 min. This showed that bacteriocin Lac-B23 had good thermal stability, but activity was susceptible to high-temperature (121°C) sterilization. In addition, the activity of bacteriocin Lac-B23 was stable between pH 2.0 and 12.0, and it was not sensitive to papain and pepsin. Nevertheless, antimicrobial activity was completely lost after treatment with trypsin, proteinase K, and proteinase E, thereby, indicating that these three could completely destroy the active site of bacteriocin Lac-B23.

### Effect of Metal Ion, Ethyl Alcohol, and Sodium Chloride on Bacteriocin Activity

Antimicrobial activity of bacteriocin Lac-B23 was completely or partially inactivated by Cu^2+^, K^+^, Ca^2+^, Zn^2+^, and Mg^2+^ treatments, whereas it was increased by Fe^2+^ and Mn^2+^ treatments (**Figure 6C**). Ethyl alcohol also had a positive effect on antimicrobial activity. Results showed that antimicrobial activity of bacteriocin rapidly increased when ethyl alcohol content increased from 2 to 12% (**Figure 6D**). We propose that the increase in activity due to Fe^2+^, Mn^2+^, and ethyl alcohol might be due to their synergistic effect with bacteriocin. Overall, it implied that bacteriocin combined with other food antiseptic technology would be more effective in food preservation. On the contrary, bacteriocin activity was inhibited by the treatment of sodium chloride (concentration between 2 and 12%) (**Figure 6E**), possibly because sodium chloride partially destroys the active structure of bacteriocin Lac-B23. However, the actual mechanism remains to be clarified.

## Conclusion

In this study, a bacteriocin-producing *L. plantarum* J23 was screened, out of 300 strains, from Chinese traditional fermented milk. Bacteriocin Lac-B23 was purified by a three-step purified method and its molecular mass was determined to be approximately 6.73 kDa. Moreover, bacteriocin Lac-B23 exhibited good thermal and pH stability, and was sensitive to trypsin, proteinase K, and proteinase E. Its activity could be improved by adding Fe^2+^, Mn^2+^, and ethyl alcohol; however, its antimicrobial activity was inhibited by Cu^2+^, K^+^, Ca^2+^, Zn^2+^, Mg^2+^, and sodium chloride. Mass spectrum identification and characterization of bacteriocin suggested bacteriocin Lac-B23 as a novel bacteriocin, with potential application prospects in the food industry.

## Author Contributions

JZ and YY performed majority of the experiments (strain isolation and partial purification) and contributed equally to this work. HYa and YB performed bacteriocin identification and fermentation. LZ and LA performed the experiments to determine the effect of heat, pH, enzymes, metal ion, ethyl alcohol, and sodium chloride on bacteriocin activity. HYi and XH are the corresponding authors, and supervised the work and contributed to the manuscript. All authors have read the manuscript and approved its submission to this journal.

## Conflict of Interest Statement

The authors declare that the research was conducted in the absence of any commercial or financial relationships that could be construed as a potential conflict of interest.
